# The effects of feeding and starvation on antioxidant defence, fatty acid composition and lipid peroxidation in reared *Oncorhynchus mykiss* fry

**DOI:** 10.1038/s41598-021-96204-y

**Published:** 2021-08-18

**Authors:** Hatayi Zengin

**Affiliations:** Department of Mathematics and Science Education, Faculty of Education, Cumhuriyet University, 58140 Sivas, Turkey

**Keywords:** Biochemistry, Physiology, Biomarkers

## Abstract

The effects of feeding and starvation have been studied with respect to oxidative stress and enzymatic antioxidant activities in the whole body of 4 cm rainbow trout fry *Oncorhynchus mykiss* (Walbaum 1792). The experiment was conducted for 28 days. The selected biomarkers for the study were determined, including non-enzymic scavengers glutathione (GSH), oxidized glutathione (GSSG) and malondialdehyde (MDA) contents and a number of enzymes are known to have major antioxidant activity, such as activities of süperoksit dismutaz (SOD), catalase (CAT), glutatyon peroksidaz (GSHpx), glutatyon Redüktaz (GR) and Glutatyon-*S*-Transferaz (GST). There is an endogenous cellular glutathione pool which consists of two forms of glutathione, i.e. the GSH and the GSSG. Oxidative damage was measured by the formation of MDA as an indication of lipid peroxidation. The activities of SOD in 14th and 28th day and the activity of CAT in 14th day were increased significantly during the 28 days of starvation. GSHpx and GR activities in starved fry decreased significantly in 28th day. GST activity in all starved fry showed the most significant increases the period of 28 days starving. The highest ΣSFA (Total Saturated Fatty Acid) content was obtained from 28 day starved fry. In starved fry, there was an apparent preference in utilization of C18:1n-9 than in the fed fry. In both starved and fed fry, C16:1n-7 was preferentially kept during the same period. Fry kept 28 days under starvation conditions exhausted C15:0, C17:0, C18:3n-6, C22:0, C24:0. They utilized less C20:5n-3 acid and conserved strongly C22:6n-3 acid. Concentrations of C20:5n-3, C22:5n-3, C22:6n-3 and total n-3 fatty acids significantly increased and C18:3n-3 significantly decreased in the whole body of starved fry during starvation period. A significant increase in the concentrations of C22:5n-3 and C22:6n-3 was determined in the fed fries in the last 2 weeks. Fat-soluble vitamins, cholesterol, stigmasterol and β-sitosterol levels were also determined in the same period of *O. mykiss* fry.

## Introduction

As it is in mammals, antioxidant defense mechanisms of fish is also composed of enzyme systems and antioxidant substances with a low molecular weight^1^. Such defense mechanisms include direct or indirect interactions between the enzymes and free-radical scavenging substances again with a low molecular weight. Similar with the antioxidants, scavenging substances also react against reactive oxygen species and oxidative degradation reactions like lipid peroxidation^2^. Such cellular mechanisms minimize or prevent adverse impacts of reactive oxygen species through antioxidant enzymes^3^ including superoxide dismutase (SOD), catalase (CAT), glutathione reductase (GR), glutathione peroxidase (GSHpx) and glutathione *S*-transferase (GST)^4^. Antioxidant enzymes are able to remove reactive oxygen species and reduce or prevent lipid peroxidation reactions.

SOD enzyme discovered by McCord and Fridovich^5^ was proved to have significant effects on reactive oxygen species. SOD reacts against ROS and catalyze the reaction converting O_2_ˉ into O_2_ and H_2_O_2_ further converted into water with CAT or GSHpx^6^. It was also reported thatGST enzyme couldprevent toxicity of H_2_O_2_ and organic peroxides through GSH and GR participated in recycle of GSH molecules^7–11^. H_2_O_2_ is also cleaved into water and molecular oxigen by CAT^12^. GST was reported to play a significant role in oxidative stress cases through inhibiting toxicity of lipid peroxides and reducing ROS formation with GSHpx activity^13^.

If the ROS formation levels exceed the antioxidant capacity of the cells, then an oxidative stress develops. Pollutants, oxidized foodstuffts and lack of antioxidant enzymes may result in oxidative stress and biological systems have limited capacity to cope to inhibit or reduce reactive oxygen and nitrogen species. Antioxidant enzymes repond to oxidative stress through stabilization and relocation of electrons of the free radicals and converting protonic hydrogen into unpaired oxygen electron^14,15^. The GSH, retinoic acid (vitamin A), tocopherol (vitamin E) and ascorbate (vitamin C)-like antioxidants with a low molecular weight have significant contributions to antioxidant capacity of the cells. Among these antioxidants, GSH is abundantly available and is able to reach against ROS directly. The GR can restore the balance between GSH and oxidized glutathione (GSSG). Therefore, GSH, GSSG and GR activities are commonly used as an indicators or biomarkers of oxidative stress in fish^12^.

Besides enzymatic antioxidants, fish have non-enzymatic antioxidants including retinoic acid, tocopherol. They are considered as the primary non-enzymatic antioxidants of the cells^16–18^. Retinoic acid with a free alcohol form can supress singlet oxygen and improve antioxidant enzyme activity^8,19,20^. Tocopherol has a scavenging effect on free radicals and thus tocopherol prevents lipid peroxidation in cell membranes. Dietary lipid is indispensable for maintaining physiological processes that result in normal growth and resistance to disease in fish.

Oxidative stress reduces growth and survival rates and result in anemia, muscular dystrophy and liver degeneration in fish species^21^. Resultant reactive oxygen species (ROS) may have detrimental effects on DNA and enzyme activity and bring on structural protein degradation and unsaturated lipid peroxidation, ultimately ending up with pathologies and abnormal development^1^. Portner and Farrell^22^ indicated that deprivation for food decrease energy expenditure and oxygen consumption and resultant hypoxia than generate an oxidative stress. Pascual et al.^23^ reported reduced GSH concentrations of gilt-head bream (*Sparus aurata*) with food deprivation.

Since salmonoids are able to use enzymatic and non-enzymatic mechanisms as an antioxidative defense system, they have been commonly used in oxidative stress studies. Food restriction, so called as food deprivation, have various negative effects on antioxidative defense mechanisms of fish species^24^. Takeuchi and Watanabe^25^ reported that excessive feeding of *O. mykiss* also resulted in poor growth rates and low feed conversions. Bell and Sargent^26^ indicated that fish size significantly influenced DHA synthesis of *O. mykiss*. Since excessive feeding and starvation both constitute a stress factor, cellular responses mechanism against both stressors should be well elucidated for sustainable management of aquacultural species, especially of economically important species like *Oncorhynchus mykiss* (Walbaum 1792)^27^. There is still a need for further studies for better comprehension of correlations between oxidative stress—antioxidant defense mechanisms and lipid metabolisms of fish fries under feeding and starvation conditions. Therefore, present study was designed to investigate the effects of feeding and starvation oxidative stress and antioxidative status of *Oncorhynchus mykiss* fry.

## Materials and methods

### Fish and experimental set up for the feeding and starving experiment

The *O. mykiss* were obtained from local and commercial fish farm Yeşilova in Zara (Sivas–Turkey). Eggs and sperm samples used in the present study were obtained from three females and males aged 4 and 3 years, respectively. Mature *O. mykiss* were artificially spawned; the eggs were fertilized by conventional procedures and immediately transported to a hatchery. Eggs were fertilized in February and water conditions were as follows: the water temperature was 9.7 °C during embryogenesis, 10.9 °C during yolk-sac larvae in March and was 12.1 °C during 4 weeks feeding and starvation period of development in April. pH and oxygen level of the water varied between 7.4–7.6 and 8.5–8.3 mg/L respectively from February to April. Hatching occurred 35 days after the fertilization and the yolk-sacs were completely exhausted 19 days posthatching after the embryonic development. When the *O. mykiss* larvae finished their endogenous feeding and the larvae being at their free-swimming stage^28,29^. Larvae no longer depend on a yolk-sac and can feed themselves. ‘Fry’ in this paper refers to 4 weeks later from the free-swimming stage, to a total length of 4.00 ± 0.05 cm, and to an average body weight of 0.87 ± 0.05 g in the control group. *O. mykiss* fry were divided into two groups as a fed group and the starved group. The first group was fed for 7 days, 14 days, 21 days and 28 days, and the second group was starved for 7 days, 14 days, 21 days and 28 days. All the samples of fed and starved fry *O. mykiss* (0.87 ± 0.05 g × 3 replicates) received were frozen in liquid nitrogen and stored at − 80 °C prior to the preparation of the homogenate.

The pool in which the fry fish were reared had a flow-through water supply originating from an underground natural spring. The water flow rate was 26 L/min.

### Analytical methods: determination of enzyme activities

Whole body of fed fry and starved fry *O. mykiss* (0.87 ± 0.05 g × 3 replicates) were homogenized in ice-cold buffer (20 mM phosphate buffer pH7.4, 1 mM EDTA and 0.1% Triton X-100). Homogenates were centrifuged at 30,000×*g* for 30 min. After centrifugation, debris was removed and it was analyzed for fatty acids and vitamins. The supernatant was collected and frozen at − 80 °C until analysed. The resultant supernatants were used directly for enzyme assays.

Superoxide dismutase (SOD) (EC 1.15.1.1) activity was assayed in terms of its ability to inhibit the oxygen-dependent oxidation of adrenalin (epinephrine) to adenochrome by xanthine oxidase plus xanthine^30^. The reaction was followedat 480 nm and one unit of SOD activity is defined as the amountof the enzyme causing 50% inhibition of the rate of adenochrome production at 26 °C. Solutions used in SOD activity measurement were made fresh daily. The assays were run by adding to the cuvette sequentially 0.05 M potassium phosphate buffer pH7.8/0.1 mM EDTA, 100 μl adrenaline, 100 μl xanthine and 200 μl sample. The reaction was then initiated by adding 20 μl xanthine oxidase.

Catalase (CAT) (EC 1.11.1.6) activity was measured by following the reduction of H_2_O_2_ at 30 °C and 240 nm using the extinction coefficient 0.04 mM^−1^/cm^31^. Immediately before assay, a stock solution was prepared. The quartz assay cuvette contained 50 μl sample solution in a final volume of 250 μl containing 67 mM phosphate buffer pH 7.0 and 20 mM H_2_O_2_. One unit of CAT represents the amount of enzyme that decomposes 1 μmol of H_2_O_2_ per minute.

Glutathione peroxidase (GSHpx) (EC 1.11.1.9) was assayed by following rate of NADPH oxidation at 340 nm by the coupled reaction with GR^32^. The GSSG generated by GSHpx was reduced by GR and NADPH oxidation was monitored at 340 nm. The quartz assay cuvette containing the reaction mixture which consisted of 50 Mm potassium phosphate buffer (pH 7.1), 1 mM EDTA, 3.6 mM GSH, 3.6 mM sodium azide, 1 IU/mL glutathione reductase, 0.2 mM NADPH and 0.05 mM H_2_O_2_. Moreover, 0.05 mM cumene hydroperoxide was used as substrate instead of H_2_O_2_. Sample was added and specific activities were determined using the extinction coefficient of 6.22 mM^−1^/cm.

Glutathione reductase (GR)^32^ (EC 1.6.4.2) activity was determined by the oxidation of NADPH at 340 nm using the extinction coefficient 6.22 mM^−1^/cm. Reaction mixture in quartz assay cuvette consisted of 0.1 M potassium phosphate buffer (pH 7.2), 2 mM EDTA, 0.63 mM NADPH and 0.15 mM GSSG. The reaction was initiated by the addition of the sample.

Glutathione *S*-transferase (GST)^32^ (EC 2.5.1.18) activity was measured at 340 nm with 1 mM 1-chloro-2,4-dinitrobenzene (CDNB) and 1 mM GSH in 100 mM potassium phosphate buffer, pH 6.5. The quartz assay cuvette containing 100 mM potassium phosphate buffer pH 6.5. 100 mL GSH and 100 mL CDNB were prepared and the reaction was initiated by the addition of 50 mL sample. Specific activities were determined using an extinction coefficient of 9.6 mM^−1^/cm.

### Vitamin, total protein, glutathione (GSH), oxidized glutathione (GSSG) and malondialdehyde (MDA) content of samples

The levels of Vitamin A, D, E, K and Cholesterol, Stigmasterol and β-sitosterol were analysed by Shimadzu full VP series HPLC according to the method of Katsanidis and Addis^33^. Total protein, GSH, GSSG and malondialdehyde (MDA) levels were spectrophotometrically measured. They were assayed at 750 nm according to the method of Lowry et al.^34^ with bovine serum albumin as a standard, 412 nm according to the method of Teare et al.^35^ and 532 nm according to the method of Salih et al.^36^ respectively.

### Fatty acid analyses

Total lipid contents of starved *O. mykiss* fry were extracted after homogenization in 3:2 (v/v) hexane isopropanol mixtures according to procedures described by Hara and Radin^37^. All solvents contained 0.01% butylated hydroxytoluene as an antioxidant. Fatty acid methyl esters were prepared from total lipid by acid catalyzed transmethylation at 55 °C for 15 h according to method of Christie^38^. They were analysed in a GC-17A Shimadzu gas chromatograph equipped with SPTM-2380 fused silica capillary column 30 m × 0.25 mm × 0.2 μm film thickness.

### Data analysis

The statistical analyses were performed using commercial statistical software (SPSS 15.0) for Windows. All analytical determinations were performed in triplicate and the mean values (mean ± SE) were reported. All data were statistically compared by one way variance analysis (ANOVA) and comparisons between means were performed with Tukey’s test. The homogeneity of variance assumption was checked with the Levene test and it was concluded that the subgroups in all comparisons satisfied the homogeneity of variance assumption at 0.01 significance level. Differences between means were reported as insignificant if *P* > 0.05, significant if *P* < 0.05, more significant if *P* < 0.01 and most significant if *P* < 0.001^39^.

Shapiro–Wilk test is a more suitable method for small sample sizes (n < 50) in normal distribution analyses. For larger samples (n ≥ 50), Kolmogorov–Smirnov test is used. When *P* > 0.05 as a result of the Shapiro–Wilk test, the hypothesis is accepted and the data is called normally distributed^40^. The normal distribution analysis of the data of our study was performed with the Shapiro–Wilk test in SPSS. According to the test results, the significance level of the data was found to be greater than 0.05 (*P* > 0.05).

### Ethical statement

All stages of this fish experiment has been approved by Animal Experiments Local Ethics Committee (HADYEK) at Sivas Cumhuriyet University. All experimental procedures were conducted in accordance with the guidelines of the ethics committee and the regulations in the manusript.

## Result

### Oxidative stress in* Oncorhynchus mykiss *fed and starved fry

#### Changes in SOD, CAT, GSHpx, GR and GST activities in *Oncorhynchus mykiss* fed and starved fry

The specific activities of SOD, CAT, GSHpx, GR and GST of the antioxidation system in fed and starved fry of *O. mykiss* over a period of 28 days feeding and starving are shown in Table 1.

**Table 1:** Changes in antioxidant enzymes, superoxide dismutase (SOD), catalase (CAT), glutathione peroxidase (GSHpx), glutathione reductase (GR) and glutathione *S*-transferase (GST) activities in 7, 14, 21 and 28 days fed and starved fry of *Oncorhynchus mykiss*.*

	7 day fed fry	14 day fed fry	21 day fed fry	28 day fed fry
SOD (U/g)	7.46 ± 0.16^a^	8.52 ± 0.28^b^	10.07 ± 0.77^d^	9.37 ± 0.54^c^
CAT (µg/g/1 min)	454.02 ± 10.11^a^	298.92 ± 25.54^c^	310.88 ± 12.33^c^	275.93 ± 13.45^c^
GSHpx (U/g/1 min)	84.55 ± 2.72^a^	83.97 ± 3.03^a^	110.25 ± 2.79^c^	50.24 ± 2.25^d^
GR (U/g/1 min)	23.16 ± 1.26^a^	14.51 ± 0.75^c^	6.49 ± 0.87^d^	11.06 ± 1.02^c^
GST (µg/g/1 min)	259.66 ± 12.45^a^	188.00 ± 5.77^b^	176.33 ± 6.36^b^	77.95 ± 3.31^d^

	7 day straved fry	14 day straved fry	21 day straved fry	28 day straved fry
SOD (U/g)	5.23 ± 0.27^a^	9.31 ± 0.38^d^	7.49 ± 0.20^c^	11.68 ± 0.46^d^
CAT (µg/g/1 min)	336.36 ± 4.41^c^	558.35 ± 2.16^d^	347.88 ± 5.51^c^	216.21 ± 12.17^d^
GSHpx (U/g/1 min)	31.15 ± 0.82^a^	27.13 ± 1.89^a^	19.38 ± 0.75^d^	11.80 ± 0.52^d^
GR (U/g/1 min)	2.41 ± 0.11^a^	2.41 ± 0.08^a^	1.94 ± 0.06^c^	1.31 ± 0.03^d^
GST (µg/g/1 min)	117.57 ± 2.56^d^	143.67 ± 8.87^d^	135.84 ± 6.58^d^	144.39 ± 5.29^d^

*Each value is the mean ± S.E. (standard error) of 3 repetitions. Superscripts after values in a same line with different letters represent significant difference.

^a^Values of *P* > 0.05 is not statistically significant.

^b^Values of *P* < 0.05 is statistically significant.

^c^Values of *P* < 0.01 is statistically more significant.

^d^Values of *P* < 0.001 is statistically most significant.

In *O. mykiss* fed fry, the activity of the primary radical scavenging enzyme, SOD showed its highest value in the last 2 weeks, while in *O. mykiss* starved fry SOD activity showed its highest value in the second week and the fourth week. However, SOD activities both in fed and starved fry showed their lowest values in the first week (7 days fries).

Regarding the CAT activity in 7 days and 21 days *O. mykiss* starved fry, it was more significantly (*P* < 0.01) low according to 14 days starved fry and it was more significantly (*P* < 0.01) high according to 28 days starved fry. But the CAT activity was the most significantly (*P* < 0.001) high in 14 days starved fry and the most significantly (*P* < 0.001) low in 28 days starved fry. CAT activity in *O. mykiss* fed fry showed a more significant (*P* < 0.01) decrease in 14 days, 21 days and 28 days fed fry. A statistically significant change was not determined from day 14 to 28.

GSHpx activities in both *O. mykiss* fed and starved fry in the last week were the most significantly (*P* < 0.001) lower than the other developmental stages. But the highest activity of GSHpx was observed in 21 days fed fry. In the first 2 weeks, no significant (*P* > 0.05) change in GSHpx activity was detected in both fed and starved fry.

The enzyme with the lowest activity in the fed and starved fry was the GR enzyme in the starved fry. The GR activity in 7 day fed fry had the highest value with a value of 23.16 U/g/1 min and in 28 day starved fry had the lowest value with a value of 1.31 U/g/1 min. The lowest GR aktivity in fed fries was observed in 21 days fed fry with a value of 6.49 U/g/1 min.

GST activity in all *O. mykiss* starved fries showed its highest value throughout the period of 28 days starving but it its lowest (*P* < 0.001) value occured in *O. mykiss* fed fry in the last week. Oxidative stress and antioxidant defence markers and the role of each enzyme studied were given in Table 2.

**Table 2:** Oxidative stress and antioxidant defence markers. The role of each enzyme studied.

Enzymatic antioxidants^41–44^	
Superoxide dismutase (SOD, E.C. 1.15.1.1)	SOD converts superoxide anions (O_2_·−) into H_2_O_2_
Catalase (CAT, E.C. 1.11.1.6)	CAT converts H_2_O_2_ to molecular oxygen (O_2_), and water (H_2_O)
	CAT and SOD are the major defences against ROS
Glutathione peroxidase (GSHpx, EC 1.11.1.9)	GSHpx are a large family of diverse isozymes that use GSH to reduce H_2_O_2_ and organic and lipid hydroperoxides
Glutathione reductase (GR, EC 1.6.4.2)	GR acts to maintain levels of reduced glutathione, and glutathione-*S*-transferase (GST). Some isoenzymes of which may metabolize lipid hydroperoxides
Glutathione-*S*-transferase (GST, EC 2.5.1.18)	The oxidised glutathione (GSSG) is reduced back to GSH by the enzyme glutathione reductase (GR) which uses NADPH as the electron donor
Reduced glutathione (GSH)	GSH is abundantly available and is able to reach against ROS directly. It plays a role in the rescue of cells from apoptosis; depletion of GSH is concomitant with the starting of apoptosis
	GSH scavenges hydroxyl radical and singlet oxygen directly, detoxifying H_2_O_2_ and lipid peroxides by the catalytic action of glutathion peroxidase
Oxidized glutathione (GSSG)	GSSG is reduced back to GSH by the enzyme glutathione reductase
NADPH	Glutathione reductase uses NADPH as the electron donor
H_2_O_2_	The increased SOD activity could be linked to the increased H_2_O_2_ production
Reactive oxygen species (ROS)	ROS may result in oxidative damage to key biological molecules, such as lipids
ROS include	The superoxide anion radical (O_2_^−·^), hydrogen peroxide (H_2_O_2_) and the highly reactive hydroxyl radical, HO^.^
Malondialdehyde (MDA)	Lipid peroxidation level was determined by malondialdehyde. MDA can react with DNA bases Guanine, Adenine, and Cytosine

Enzymatic antioxidant scavenging systems that maintain endogenous reactive oxygen species (ROS) at relatively low levels.

#### Changes in total protein, GSH, GSSG and MDA levels in *Oncorhynchus mykiss* fed and starved fry

The levels of total protein (mg/g), GSH (µg/g), GSSG (µg/g) and MDA (nmol/g) were examined in fed and starved fry of *O. mykiss* over a period of 28 days feeding and starving are shown in Table 3.

**Table 3:** Changes in Total Protein (Tot.Prot.), GSH, GSSG and MDA levels in fed and starved fry of *Oncorhynchus mykiss*.*

	7 day fed fry	14 day fed fry	21 day fed fry	28 day fed fry	Diet
Tot.Prot.mg/g	76.67 ± 1.60^d^	60.42 ± 2.04^b^	65.17 ± 0.80^c^	76.50 ± 1.75^d^	83.75 ± 1.59
GSH µg/g	184.48 ± 7.03^a^	261.95 ± 8.48^d^	201.71 ± 6.35^b^	216.21 ± 11.68^c^	–
GSSG µg/g	21.21 ± 1.43^a^	23.87 ± 1.94^b^	26.51 ± 4.11^c^	18.80 ± 0.42^a^	–
MDA nmol/g	149.71 ± 9.30^a^	184.79 ± 3.79^c^	190.86 ± 4.84^c^	162.04 ± 8.12^b^	–

	7 day starved fry	14 day starved fry	21 day starved fry	28 day starved fry	–
Tot.Prot.mg/g	65.67 ± 0.79^d^	49.92 ± 0.96^c^	54.83 ± 1.45^a^	65.67 ± 1.10^d^	–
GSH µg/g	216.62 ± 11.98^a^	118.23 ± 4.90^c^	139.33 ± 10.16^c^	201.05 ± 18.28^a^	–
GSSG µg/g	16.24 ± 0.42^a^	32.61 ± 2.08^c^	22.91 ± 3.49^b^	24.39 ± 0.50^b^	–
MDA nmol/g	144.65 ± 6.44^a^	221.22 ± 10.24^d^	209.45 ± 14.24^d^	155.81 ± 13.64^a^	–

* The meaning of the symbols is given under Table 1.

The highest total protein value (83.75 mg/g) was observed in the commercial feed (*P* < 0.001). Similar changes in total protein content were observed in fed fry. 7 day and 28 day fed and starved fry have the highest total protein values and 14 day and 21 day fed and starved fry have the low total protein values. It was observed that no significant change (*P* > 0.05) had occurred in the total protein content of 7 day and 28 day fed fry. Similar result was observed in 7 day and 28 day starved fry. But the amount of the total protein content of 28 days fed fry was being higher than the that of starved fry in the same developmental stage.

14 day fed fry exhibited significantly higher GSH concentration in comparison with 14 day starved fry. GSH concentration was significantly lower in 14 day starved fry. The GSH concentration in 7 day fed fry and starved fry showed opposite trends in both groups, being significantly higher in 7 day starved fry.

The difference in the GSSH concentration between 7 and 28 days fed fry was found to be statistically insignificant (*P* > 0.05). The difference in the GSSH concentration between 21 and 28 days starved fry was also found statistically insignificant (*P* > 0.05). The GSSH concentration in 21 days fed fry and in14 days starved fry exhibited statistically more significant (*P* < 0.01) high values.

The most significant (*P* < 0.001) increase in the MDA concentration was detected in 14 days and 21 days starved fry. There was no significant (*P* > 0.05) change in the MDA concentration of starved fry at the other stages of the starving period. In our study, MDA levels from 14 to 21 days fed fry remained relatively constant, but it decreased significantly (*P* < 0.05) in 28 days fed fry.

#### Changes in retinol, vitamin D_2_, vitamin D_3_, δ-tocopherol , α-tocopherol, vitamin K_1_, vitamin K_2_, cholesterol, stigmasterol and β-sitosterol levels in *Oncorhynchus mykiss* fed and starved fry

The levels of retinol (μg/g), vitamin D_2_ (μg/g), vitamin D_3_ (μg/g), vitamin K_1_ (μg/g), vitamin K_2_ (μg/g), δ-tocopherol (μg/g), α-tocopherol (μg/g), cholesterol (mg/g), stigmasterol (μg/g) and β-sitosterol (μg/g) were examined in fed and starved fry of *O. mykiss* over a period of 28 days feeding and starving are shown in Table 4.

**Table 4:** Changes in Retinol, Vitamin D_2,_Vitamin D_3_, Vitamin K_1_, Vitamin K_2_, δ-Tocopherol, α-Tocopherol, Cholesterol, Stigmasterol and β-sitosterol levels in fed and starved fry of *Oncorhynchus mykiss*.*

	7 day fed fry	14 day fed fry	21 day fed fry	28 day fed fry	Diet
Retinol (vit. A) µg/g	0.07 ± 0.001^a^	0.11 ± 0.03^b^	0.09 ± 0.001^a^	0.12 ± 0.02^b^	1.20 ± 0.08
Vitamin D_2_ µg/g	nd	nd	nd	nd	nd
Vitamin D_3_ µg/g	0.32 ± 0.06^c^	0.77 ± 0.06^d^	0.75 ± 0.08^d^	0.62 ± 0.06^d^	1.20 ± 0.08
Vitamin K_1_ µg/g	0.27 ± 0.05^b^	0.42 ± 0.04^c^	0.21 ± 0.03^b^	0.50 ± 0.07^d^	2.51 ± 0.17
Vitamin K_2_ µg/g	nd	nd	nd	nd	nd
δ-Tocopherol µg/g	0.20 ± 0.02^b^	0.27 ± 0.01^a^	0.12 ± 0.03^d^	0.17 ± 0.02^c^	0.60 ± 0.08
α-Tocopherol µg/g	29.31 ± 1.21^b^	21.92 ± 0.17^b^	16.44 ± 0.05^d^	25.01 ± 2.04^a^	6.67 ± 0.50
Cholesterol µmol/g	0.84 ± 0.04^a^	0.87 ± 0.03^a^	0.74 ± 0.01^b^	0.74 ± 0.07^b^	0.51 ± 0.01
Stigmasterol µg/g	67.48 ± 3.40^b^	82.87 ± 3.34^d^	63.09 ± 0.85^a^	72.45 ± 3.67^c^	326.91 ± 0.52
β-Sitosterol µg/g	10.47 ± 0.43^b^	18.13 ± 0.53^d^	8.02 ± 0.33^a^	7.81 ± 0.46^b^	103.69 ± 0.50

	7 day starved fry	14 day starved fry	21 day starved fry	28 day starved fry	
Retinol (vit. A) µg/g	0.08 ± 0.001^a^	0.11 ± 0.006^b^	0.16 ± 0.02^c^	0.19 ± 0.03^d^	–
Vitamin D_2_ µg/g	0.39 ± 0.01^c^	0.54 ± 0.02^a^	0.34 ± 0.01^c^	0.20 ± 0.01^d^	–
Vitamin D_3_ µg/g	0.18 ± 0.01^a^	0.52 ± 0.03^d^	0.24 ± 0.03^b^	0.20 ± 0.01^b^	–
Vitamin K_1_ µg/g	0.36 ± 0.01^c^	0.45 ± 0.03^c^	0.21 ± 0.01^d^	0.06 ± 0.001^d^	–
Vitamin K_2_ µg/g	nd	nd	nd	nd	–
δ-Tocopherol µg/g	0.18 ± 0.01^c^	0.27 ± 0.01^a^	0.19 ± 0.01^c^	0.28 ± 0.01^a^	–
α-Tocopherol µg/g	15.71 ± 0.95^d^	21.78 ± 0.66^b^	19.00 ± 0.58^c^	12.33 ± 0.92^d^	–
Cholesterol µmol/g	2.11 ± 0.04^b^	2.49 ± 0.06^c^	3.13 ± 0.05^d^	1.59 ± 0.12^d^	–
Stigmasterol µg/g	59.31 ± 1.91^a^	70.12 ± 3.51^b^	66.86 ± 1.87^b^	70.13 ± 4.02^b^	–
β-Sitosterol µg/g	10.31 ± 0.49^c^	15.09 ± 0.83^d^	12.35 ± 0.22^c^	6.82 ± 0.27^a^	–

* The meaning of the symbols is given under Table 1.

*nd* Not detected.

All parameters studied in Table 4 were high in diet and low in *O. mykiss* fry fed over a period of 28 days except for α-tocopherol and cholesterol of which the low values were noted. When retinol levels were examined throughout the 28 days, fluctuations in the retinol levels were observed for the fed fries during their feeding period (Table 4). However, unlike the fed fry, the retinol levels of starved fry showed a steady increase throughout the starving period. The statistical significance of the increments in the retinol levels of starved fries were found to be *P* > 0.05 for 7 days, *P* < 0.05 for 14 days, *P* < 0.01 for 21 days and *P* < 0.001 for 28 days (Table 4).

Vitamin D_2_ and Vitamin K_2_ could not be determined in both diet and fed fry (Table 4). Vitamin K_2_ could not be determined in also starved fry (Table 4). However, unlike the fed fry, Vitamin D_2_ was detected in starved fry. It showed its highest value in 14 days starved fry, but it decreased sharply in 28 days starved fry (*P* < 0.001). There were no significant differences in the level of vitamin D_3_ from 14 to 28 days fed fry of *O. mykiss*, but the more significant increase was observed in 14 days fed fry. Similar to 14 days fed fry, vitamin D_3_ in starved fry showed its highest value in second weeks, although the amount is less. While the vitamin K_1_ level in 28 days starved fry exhibited significantly (*P* < 0.001) low values (0.06 ± 0.001), the most significant (*P* < 0.001) increase in the vitamin K_1_ level (0.50 ± 0.07) was detected in 28 days fed fry. Oxidative stress and antioxidant defence markers and the role of each molecules studied were given in Table 5.

**Table 5:** Oxidative stress and antioxidant defence markers. The role of each molecules studied.

Non-enzymatic antioxidants^41–46^	
Retinol	Retinol supress ^1^O_2_, singlet oxygen
δ-Tocopherol, α-Tocopherol	Tocopherol is major membrane-bound lipid-soluble antioxidant responsible for protecting the polyunsaturated fatty acids in membranes against lipid peroxidation
Vitamin K_1_, Vitamin K_2_	Vitamin K has role in the blood coagulation
Vitamin D_2_, Vitamin D_3_	Vitamin D plays a role in the absorption of calcium and phosphate from the intestine and the storage of these minerals in the bone
Cholesterol	Cholesterol plays has a role in membrane fluidity but it’s most important function is in reducing the permeability of the cell membrane
	It helps to restrict the passage of molecules by increasing the packing of phospholipids
	Cholesterol can fit into spaces between phospholipids and prevent water-soluble molecules from diffusing across the membrane
	The hydrophilic hydroxyl group of cholesterol interacts with aqueous environment, whereas the large hydrophobic domain fits between C-tails of lipids
Stigmasterol	Phytosterols have been shown to compete with dietary cholesterol to be absorbed by the intestine
	Stigmasterol is a molecule that inhibits cholesterol increase and may be effective in reducing cholesterol absorption
β-Sitosterol	Lower levels of phytosterols, like those found in diets rich in plant foods, may be effective in reducing cholesterol absorption
PUFA	PUFA (Polyunsaturated fatty acid) DHA and EPA in *O. mykiss* fry during starvation were conserved
MUFA	MUFA, especially of C18:1n-9 were mostly used as a source of energy
SFA	The proportion of saturated fatty acids (SFA) was significantly influenced by the early developmental stages

Non enzymatic antioxidant scavenging systems that maintain endogenous reactive oxygen species (ROS) at relatively low levels.

δ-Tocopherol level showed the most significant (*P* < 0.001) decrease in 21 days fed fry and then the more significant (*P* < 0.01) increase in 28 days fed fry. Similar results have also been found in α-tocopherol showed the most significant (*P* < 0.001) decrease in 21 days fed fry and then the increase in 28 days fed fry. When δ-tocopherol levels in starved fries were examined throughout the 28 days, fluctuations in the δ-tocopherol levels were observed during their starving period. α-tocopherol in starved fry notably (*P* < 0.001) declined in 28 days starved fry.

When cholesterol levels were examined throughout the feeding period of 28 days, there were no significant differences from 7 to 14 days fed fry. Similarly, there were no differences in the level of cholesterol between 21 and 28 days fed fry of *O. mykiss*, but cholesterol levels showed a small statistically significant (*P* < 0.05) decrease in 21 and 28 days fed fry according to the 7 and 14 days fed fry. The starved fries had a highest cholesterol level in 21 days starved fry (3.13 ± 0.05) and a lowest cholesterol level in 28 days starved fry (1.59 ± 0.12). Cholesterol levels in the all starved fries were noteworthy when compared to the all fed fries.

The level of stigmasterol changed the most significantly from 7 days fed fry to 14 days fed fry, however it showed a dramatic decrease from 14 days fed fry to 21 days fed fry. There were no significant (*P* > 0.05) differences in stigmasterol levels from 14 to 28 days starved fries, the low value in stigmasterol levels was observed in the first week according to the remaining 3 weeks.

β-sitosterol levels showed a slight increase in 7 days fed and starved fries (*P* < 0.05; *P* < 0.01 respectively), but it increased sharply (*P* < 0.001) in 14 days fed (18.13 ± 0.53) and starved (15.09 ± 0.83) fries. β-sitosterol levels in *O. mykiss*’s fed and starved fry showed the remarkable decrease in 28 days fed (7.81 ± 0.46) and starved (6.82 ± 0.27) fries.

### Changes in fatty acid composition of* Oncorhynchus mykiss* fed and starved fry

Fatty acid compositions of *O. mykiss* fed fry and diet and starved fry from 7 to 28 days are presented in Tables 6 and 7 respectivelly.

**Table 6:** Fatty acid composition of fed fry of *Oncorhynchus mykiss* and diet.*

Fatty acids	7 day fed fry	14 day fed fry	21 day fed fry	28 day fed fry	Feed
C14:0	1.32 ± 0.06^a^	1.32 ± 0.01^a^	1.24 ± 0.01^a^	1.33 ± 0.04^a^	2.67 ± 0.11^b^
C15:0	nd	nd	nd	nd	0,30 ± 0,04
C16:0	14.35 ± 0.20^a^	14.58 ± 0.08^ab^	14.81 ± 0.09^b^	14.71 ± 0.08^ab^	18.32 ± 0.01^c^
C17:0	0.51 ± 0.03^b^	0.43 ± 0.03^c^	0.25 ± 0.02^d^	0.45 ± 0.02^bc^	0.61 ± 0.04^a^
C18:0	4.88 ± 0.13^a^	4.83 ± 0.04^a^	4.83 ± 0.05^a^	4.88 ± 0.05^a^	nd
C22:0	nd	nd	nd	nd	nd
C24:0	0.32 ± 0.01^a^	0.29 ± 0.02^a^	0.33 ± 0.03^a^	0.29 ± 0.04^a^	0.14 ± 0.01^b^
**ΣSFA**	21.38 ± 0.37^a^	21.45 ± 0.06^ab^	21.47 ± 0.02^ab^	21.65 ± 0.11^ab^	22.04 ± 0.01^b^
C16:1n-7	2.94 ± 0.06^b^	3.43 ± 0.07^a^	3.37 ± 0.01^a^	3.28 ± 0.06^b^	4.38 ± 0.04^c^
C17:1	nd	nd	nd	nd	0,46 ± 0,03
C18:1n-9	23.46 ± 0.09^b^	22.58 ± 0.06^c^	22.52 ± 0.07^c^	22.00 ± 0.04^d^	18.79 ± 0.01^e^
C20:1n-9	1.78 ± 0.01^b^	1.46 ± 0.07^c^	1.58 ± 0.03^ad^	1.50 ± 0.04^ cd^	2.12 ± 0.03^e^
C22:1	1.04 ± 0.06^b^	1.49 ± 0.03^c^	1.10 ± 0.04^ab^	1.28 ± 0.05^d^	1.29 ± 0.01^d^
**ΣMUFA**	29.23 ± 0.08^b^	28.96 ± 0.05^c^	28.56 ± 0.03^d^	28.06 ± 0.10^e^	27.04 ± 0.05f.
C18:3n-3	1.98 ± 0.05^ab^	1.96 ± 0.08^ab^	1.85 ± 0.04^b^	1.89 ± 0.02^ab^	3.78 ± 0.03^c^
C20:5n-3	2.56 ± 0.12^bc^	2.71 ± 0.05^ab^	2.46 ± 0.04^c^	2.74 ± 0.03^ab^	5.04 ± 0.05^d^
C22:5n-3	0.96 ± 0.08^ab^	1.15 ± 0.04^bc^	1.32 ± 0.06^c^	1.22 ± 0.10^c^	0.69 ± 0.02^d^
C22:6n-3	15.51 ± 0.03^a^	15.62 ± 0.04^a^	16.12 ± 0.09^b^	16.53 ± 0.18^c^	9.40 ± 0.09^d^
**Σn-3**	21.01 ± 0.20^b^	21.44 ± 0.09^bc^	21.75 ± 0.07^ac^	22.39 ± 0.15^d^	18.91 ± 0.01^e^
C18:2n-6	24.76 ± 0.29^bc^	24.88 ± 0.11^b^	24.60 ± 0.07^bc^	24.33 ± 0.24^c^	28.36 ± 0.01^d^
C18:3n-6	0.43 ± 0.06^ab^	0.41 ± 0.03^ab^	0.49 ± 0.03^b^	0.43 ± 0.01^ab^	2.41 ± 0.02^c^
C20:2n-6	0.92 ± 0.08^b^	0.82 ± 0.03^ab^	0.78 ± 0.04^ab^	0.83 ± 0.01^ab^	0.33 ± 0.00^c^
C20:3n-6	0.60 ± 0.03^a^	0.68 ± 0.02^a^	0.71 ± 0.04^a^	0.88 ± 0.04^b^	0.07 ± 0.00^c^
C20:4n-6	1.68 ± 0.08^a^	1.35 ± 0.04^bc^	1.63 ± 0.05^a^	1.44 ± 0.04^c^	0.85 ± 0.00^d^
**Σn-6**	28.39 ± 0.22^b^	28.15 ± 0.10^bc^	28.22 ± 0.02^bc^	27.91 ± 0.18^c^	32.01 ± 0.03^d^
**ΣPUFA**	49.40 ± 0.38^b^	49.58 ± 0.03^b^	49.97 ± 0.05^bc^	50.30 ± 0.03^c^	50.92 ± 0.04^d^
Σn-3/Σn-6	0.74 ± 0.01^b^	0.76 ± 0.01^bc^	0.77 ± 0.00^c^	0.80 ± 0.01^d^	0.59 ± 0.00^e^

*The meaning of the symbols is given under Table 1.

Σ: Total. ΣSFA: Total Saturated Fatty Acid.

ΣMUFA: Total Monounsaturated Fatty Acid.

Σn-3: Total n-3 Fatty Acid.

Σn-6: Total n-6 Fatty Acid.

ΣPUFA: Total Polyunsaturated Fatty Acid.

**Table 7:** Fatty acid composition of starved fry of *Oncorhynchus mykiss*.*

Fatty acids	7 day starved fry	14 day starved fry	21 day starved fry	28 day starved fry
C14:0	1.23 ± 0.05^ab^	0.93 ± 0.06^c^	0.98 ± 0.01^ cd^	1.02 ± 0.03^ce^
C15:0	nd	nd	nd	nd
C16:0	14.22 ± 0.06^a^	14.45 ± 0.08^ab^	14.62 ± 0.08^b^	16.08 ± 0.11^c^
C17:0	nd	nd	nd	nd
C18:0	5.06 ± 0.04^a^	5.80 ± 0.08^b^	5.63 ± 0.20^b^	6.82 ± 0.09^c^
C22:0	nd	nd	nd	nd
C24:0	0.33 ± 0.01^a^	nd	nd	nd
**ΣSFA**	20.85 ± 0.10^b^	21.18 ± 0.09^ab^	21.24 ± 0.28^ab^	23.91 ± 0.16^c^
C16:1n-7	3.24 ± 0.05^ac^	2.56 ± 0.06^b^	3.06 ± 0.07^c^	3.98 ± 0.06^d^
C17:1	nd	nd	nd	nd
C18:1n-9	23.25 ± 0.13^b^	22.50 ± 0.16^c^	22.57 ± 0.24^c^	20.39 ± 0.08^d^
C20:1n-9	1.64 ± 0.09^a^	1.32 ± 0.07^b^	1.29 ± 0.04^b^	1.28 ± 0.04^b^
C22:1	1.51 ± 0.05^b^	0.86 ± 0.01^c^	0.74 ± 0.05^c^	0.56 ± 0.04^d^
**ΣMUFA**	29.64 ± 0.20^a^	27.24 ± 0.06^b^	27.66 ± 0.22^b^	26.22 ± 0.11^c^
C18:3n-3	1.94 ± 0.04^a^	1.45 ± 0.05^b^	1.53 ± 0.07^bc^	1.67 ± 0.05^c^
C20:5n-3	3.14 ± 0.06^b^	2.81 ± 0.04^a^	3.99 ± 0.07^c^	4.91 ± 0.02^d^
C22:5n-3	1.08 ± 0.05^a^	1.49 ± 0.14^bc^	1.35 ± 0.05^b^	1.59 ± 0.08^bc^
C22:6n-3	17.38 ± 0.02^b^	21.28 ± 0.07^c^	20.21 ± 0.50^d^	20.81 ± 0.10^ cd^
**Σn-3**	23.54 ± 0.12^b^	27.03 ± 0.04^c^	27.08 ± 0.39^c^	28.98 ± 0.10^d^
C18:2n-6	22.37 ± 0.07^b^	20.53 ± 0.10^c^	19.89 ± 0.10^d^	17.16 ± 0.08^e^
C18:3n-6	0.31 ± 0.01^a^	0.30 ± 0.02^a^	0.31 ± 0.03^a^	-
C20:2n-6	0.86 ± 0.07^ab^	0.89 ± 0.05^ab^	0.93 ± 0.07^a^	0.73 ± 0.02^b^
C20:3n-6	0.69 ± 0.06^a^	0.70 ± 0.06^a^	0.65 ± 0.06^a^	0.65 ± 0.01^a^
C20:4n-6	1.74 ± 0.04^a^	2.13 ± 0.07^b^	2.25 ± 0.07^bc^	2.35 ± 0.07^c^
**Σn-6**	25.97 ± 0.02^a^	24.54 ± 0.16^b^	24.03 ± 0.06^c^	20.89 ± 0.12^d^
**ΣPUFA**	49.51 ± 0.11^b^	51.58 ± 0.13^c^	51.11 ± 0.44^c^	49.87 ± 0.15^b^
**Σn-3/Σn-6**	0.91 ± 0.01^b^	1.10 ± 0.01^c^	1.13 ± 0.01^c^	1.39 ± 0.01^d^

*The meaning of the symbols is given under Table 6.

It was noted that on the investigation of the fatty acid composition of the commercial feed used in the feeding of the fish, C18:0 and C22:0 were not present (Table 6). C15:0, C17:0, C22:0, (heptadecenoic acid) C17:1 were not determined in the all starved fry. Among all groups of starved fries (7, 14, 21 and 28-day starved), lignoceric acid (C24:0) was determined only in 7 day starved fry, whereas C18:3n-6 was determined in all groups but 28-days starved fry (Table 7).

Tetradecanoic acid (myristic acid) (C14:0) and hexadecanoic acid (palmitic acid) (C16:0) were determined to be in higher percentage in the feed than in the fed fry. The level of C16:0 (18.32%) was high in the feed. The increase of this fatty acid in the starved fish was noteworthy when compared to the fish fed with this fatty acid. ΣSFA showed a significant (*P* < 0.05) and steady increase over the 28-day starving period due to increase in the content of the most abundant saturated fatty acid, C16:0 and C18:0. ΣSFA were at a minimum (21.38%) at 7 days fed fry and a maximum (21.65%) at 28 days fed fry of *O. mykiss*. There were no significant (*P* > 0.05) differences in ΣSFA content from 7 to 28 days fed fry. The most abundant fatty acid in SFAs was C16:0 in all cases.

However, in the case of total monounsaturated fatty acid (ΣMUFA), they were at a minimum (26.22%) at 28 days starved fry and at a maximum (29.64%) at 7 days starved fry of *O. mykiss*. The percentages of the ΣMUFA increased significantly (*P* < 0.05) at 7 day starved fry due to increase in the content of the most abundant unsaturated fatty acid, C18:1n-9. According to 7 day starved fry, a statistically significant (*P* < 0.05) decrease at 14 days starved fry and a statistically significant (*P* < 0.05) increase at 28 days starved fry was observed in the percentages of C16:1n-7. ΣMUFA were at a minimum (28.06%) at 28 days fed fry and reached a maximum (29.23%) at 7 days fed fry of *O. mykiss*. The percentages of the ΣMUFA increased significantly (*P* < 0.05) at 7 days fed fry due to increase in the content of the most abundant unsaturated fatty acid, C18:1n-9.

Throughout the feeding periods, total polyunsaturated fatty acids (ΣPUFA) were at a maximum (50.30%) at 28 days fed fry and at a minimum (49.40%) at 7 days fed fry of *O. mykiss*, mainly composed of the increased percentage of C18:2n-6, C18:3n-3, C20:4n-6, C20:5n-3, and C22:6n-3. Throughout the starving periods, total polyunsaturated fatty acids (ΣPUFA) were at a maximum (51.58%) at 14 days starved fry and at a minimum (49.51%) at 7 days starved fry of O. mykiss, mainly composed of the increased percentage of C18:2n-6, C18:3n-3, C20:4n-6, C20:5n-3, C22:5n-3 and C22:6n-3.

The amount of Σn-3 fatty acids was significantly higher in 28 day fed and starved fry (22.39% and 28.98% respectively) and the amount of Σn-6 fatty acids was significantly lower in 28 day fed and starved fry (27.91% and 20.89% respectively) than the other feeding and the starvation stages.

## Discussion

At the end of the feeding and starvation experiment, determination of the antioxidant enzyme activity changes in fed and starved fry is important in monitoring the physiological conditions of fries. Generally, most parameters evaluated in this study, followed an increasing tendency during starvation; this increase is statistically significant from the first days of experiment at the end of both feeding and starving periods. Various authors had earlier posited that the radicals are small molecules/ions that are reactive with small activation energies and short lifetimes. The small size makes it possible for many of them to penetrate cell membranes and starvation induced the formation of free radicals, which react with some cellular components such as membrane lipids and produce lipid peroxidation products^22,47–53^.

It is remarkable that SOD activity is highest in the 28 day starved fry (Table 1). The increased SOD activity could be linked to the increased H_2_O_2_ production. It is known that SOD value can be believed to be a more reliable and expressive set of oxidative stress indicators. High SOD activity in the whole body of 28 day starved fry might be a consequence of free radicals derived from oxidation of lipids^54–57^.

Besides, the higher CAT activities also suggest the fries were subjected to greater oxidative stres in 14 day starved fry. CAT activity showed the highest value (558.35 µg/g/1 min) in 14 day starved fry compared to 14 day fed fry. CAT activity, found mainly in peroxisomes, is associated with elevated concentrations of H_2_O_2_. These results agree with many other works^58–62^.

Significant (*P* < 0.001) decreases were observed in GSHpx activities of both 28-day-fed and starved fry, but 21-day-fed fry (Table 1). Such decreases were mainly attributed to adaptive down-regulation of GSHpx enzyme by lower free-radical levels. GSHpx CAT enzyme activities could be regulated by H_2_O_2_, CAT is associated with high and GSHpx with low H_2_O_2_ levels. Present findings revealed that current fries required greater PUFA levels at early growth stages to promote cell division for development of tissues and organs. On the other hand, high PUFA levels may increase the risk of oxidative stress with detrimental effects on cell membranes^63,64^.

In NADPH dependent reactions, GSH is generally reformed by GR. NADPH is generated by multiple enzymatic redox reactions and such reactions are distinctively synchronized with nutrient supply^65,66^. Limitations on nutrient supply reduce NADPH availability, then limit GR activity. Such a case then reflects in starved fish as impaired GR activity and consequently reduced GSH regeneration. Food deprivation was reported to result in depletion of endogenous GSH pool in fish species^9,66^. In present study, starvation yielded highly significant (*P* < 0.01) decreases in GSH contents of 14 day and 21 day starved *O. mykiss* fries, indicating lower resistance of starved fries against oxidative stress. On the other hand, greater GSH levels were seen in fed fries, except for 7-day fed fries.

The most significantly increased GST activities were observed in all starved fries, most significant difference was observed in 28-days starved fries and fed fries: was lower when compared to 28 day starved fry. In the present study, data obtained in starved fries demonstrated that strong responses were seen in oxidative stress which were key biomarkers in separating starved fries from fed fries.

Antioxidant enzymes were analyzed and significant increases in SOD and GST activities were found in parallel with starvation except for 21 day starved fry; however GSHpx and GR activities decreased during the starvation period in starved fry. But the highest activity among all enzymes found in fed and starved fries was determined in CAT as 558 µg/g/1 min in 14 days starved fries.

Protein levels are generally used to designate mobilization of proteins throughout the period of starvation. Significantly lower total protein levels were observed in 14 and 21-day starved fry and such low levels indicate the role of proteins in *O. mykiss*. Present findings on protein levels well-comply with the findings of previous studies reporting quite low total protein levels for starved fish fry^67–70^.

In present study, significant increases were observed in GSH levels of fed *O. mykiss* fry for 14, 21 and 28 days and the greatest GSH level was observed in 14-day feeding (Table 3). However, significant decreases were observed in GSH levels of *O. mykiss*-starved fry for 14 and 21 days. Such decreases greatly reduced the tissue defense mechanism against reactive oxygen species. Oxidative stress was also designed by cellular GSSH load. GSSH is generally reduced to GSH by GR through NADPH-dependent reactions. NADPH of these reactions is generated through multiple redox enzymatic reactions and such reactions are mostly dependent on food source^66^.

Jackson et al.^71^ reported that DNA base and lipid hydroperoxides were reduced by GST, thus GST prevented lipid peroxidation and resultant DNA damage. Increased GST activity improved detoxification of MDA, a toxic product of lipid peroxidation process^23^. Present findings on GST activities comply with the findings of Jackson et al.^71^ and Pascual et al.^23^ reporting remarkable GST activity of 14, 21 and 28-day starved fry and MDA levels of 14 and 21-day starved fry. MDA levels designate the lipid peroxidation levels, thus commonly used as an indicator of oxidative stress^72,73^.

Thus, the highest MDA content resulted in decreased levels of total PUFA, particularly linoleic acid (C18:2n-6) in 14, 21 and 28 days starved fry. Based on MDA levels, this results clearly showed that prolonged starvation led to oxidative stress, with starved *O. mykiss* fry showing increase in MDA with respect to the 7 day starved fry. A study with sea bream (*Sparus aurata*) evaluated the influence of prolonged starvation on MDA levels, and a significant increase of this metabolite has been reported^23^. MDA levels increased at the second and third week and GSSG levels remained high in the last 3 weeks in starved fish, but the MDA level in the last week returned to normal values when the fry readapted to the first week conditions.

A second line of defence is established by antioxidants, which can be provided only by nutritional supplements^74^, such as ascorbate, tocopherol, ubiquinones, and β-carotenes. Among the antioxidant nutrients, tocopherols; δ-tocopherol and α-tocopherol are the major membrane-bound lipid-soluble antioxidant responsible for protecting the polyunsaturated fatty acids in membranes against lipid peroxidation. If GSH enzymatically regenerates tocopherol from its one electron oxidation product, then the prevention of lipid peroxidation would be a secondary antioxidant effect^1,75^.

As a lipid soluble vitamin, α-tocopherol accumulates in liver and fatty tissues of animals, and it is incorporated into the structure of biomembranes^76^. Tocopherols protect lipids by scavenging peroxyl radicals without reacting in further chain-propagating steps. It helps PUFAs to maintain membrane fluidity by protecting them against peroxidation^77,78^. In this study *O. mykiss* fed fry showed decrease in α-tocopherol and δ-tocopherol levels at 21 day fed fry. Unlike fed fry a remarkable increase in α-tocopherol levels was observed in 14 and 21 day starved fry. However there were the most significant decrease in α-tocopherol level in 28 day starved fry of *O. mykiss* according to the 28 day fed fry. These results indicated that tocopherol deficiency impaired the antioxidant capacity in the starved fry. The intracellular levels of non-enzymatic antioxidants, GSH influenced the activity of the enzymatic antioxidants. As shown in Tables 3 and 4, decreased in GSH content and the tocopherol levels were not protect *O. mykiss* fry against lipid peroxidation. Decrease in tocopherol levels significantly decreased the GSH content in the 14 and 21 days starved fry. Decrease in tocopherol levels also significantly decreased the SOD (21 day starved fry) and GSHpx (21 day and 28 day starved fry) activities^65,79,80^. The starved fry had low GSH levels. These changes were coincident with the appearance of MDA shown as useful early biomarkers of oxidative stress. Results from Pascual et al.^23^ confirm this conclusion. Thus, the tocopherol level is an indispensable nutrient required to maintain normal physiological functions in fish and has been used in fish diets as an antioxidant substance to prevent the peroxidation of polyunsaturated fatty acids (PUFA) in fish oil^81^.

At sufficiently high concentrations, carotenoids can also protect lipids from peroxidative damage. Carotenoid pigments are widely distributed in nature where they play an important role in protecting cells and organisms. Carotenoids are important biological compounds that can inactivate electronically excited molecules, a process termed quenching^64,77^. Retinol is required for growth, reproduction and development of fish and must be obtained from the diet, because fish are incabaple of synthesizing the vitamin^82–84^. Steffens and Karst^85^ reported that β-carotene could be converted to retinol in *Oncorhynchus mykiss* fry.

When retinol levels were examined throughout the 28 days, fluctuations in the retinol levels were observed for the fed fries during their feeding period (Table 4). However, unlike the fed fry, the retinol levels of starved fry showed a steady increase throughout the starving period. This results showed that the retinol contents in 21 day and 28 day starved fry were higher especially than in 21 day and 28 day fed fry at the same period. There was a significant difference in the retinol levels (*P* < 0.01; *P* < 0.001), between starved fry and fed fry of *O. mykiss* (Table 4). Among nutrients, retinol and its metabolite, retinoic acid, is recognized as a highly active molecule in developmental processes, as both dietary deficiency or excess can give abnormal development and result in malformation in all species examined^86^. These non-enzymic antioxidants in *O. mykiss* fries are essential to ensure early antioxidant protection. The high levels of retinol in *O. mykiss* starved fry could provide the early antioxidant protection by stabilising free radicals produced in the developing fry as suggested by Palace and Werner^84^.

Analyses for fatty acids of present feed supply revealed that monounsaturated fatty acids (C16:1n-7 and C18:1n-9) were not used as a source of energy by fed fries. On the other hand, intensive use of C18:1n-9 and less use of C16:1n-7 (Tables 6, 7) were seen in starved fries^87^. Lipids are essential structural components of cell membranes and play a great role in cellular communication and energy storage. Primary functions of the lipids are mostly associated with early stages of teleost because teleost present quite a rate of growth with a great demand of lipids as the primary source of energy^29,88^.

Starved fries initially consumed C15:0, C17:0, C22:0 and C17:1 as a source of energy throughout the starvation period. These fatty acids were followed by C24:0 and C18:3n-6 fatty acids. Throughout the starvation period, C24:0 was commonly used during the first week and C18:3n-6 during the last week. Present findings comply with the results of Cejas et al. (2004)^89^ and Giménez et al. (2008)^90^ indicating that fatty acids were used as a source of energy especially in starvation periods during the development of fry.

The C18:1n-9 and C16:1n-7 were the major components of MUFA. Fatty acid analyses of feeding groups confirmed that fed fries mostly had long-chain MUFA including C18:1n-9 and C16:1n-7, which were also abundant in commercial feed. However, the intensive use of C18:1n-9 (*P* < 0.001) during starving period was observed in 28 days starved fry. Such an intense use of MUFA, especially of C18:1n-9 indicated that MUFA were mostly used as a source of enery. Accordingly, the fry starved for 28 days had the least C18:1n-9 level (20.39%), but the fry fed for 7 days had the greatest C18:1n-9 level (23.46%). On the other hand, significant increases were observed in C16:1n-7 levels of fries starved for 27 days (*P* < 0.05) (Abi-ayad et al. 2004)^91^. However a statistically significant (*P* < 0.05) increase in the percentages of C16:1n-7 was observed at 28 days starved fry. The value of C16:1n-7 in 28 days starved fry was being higher than the fed fry in the same developmental stage. These increases are probably the result of the synthesis of fatty acids from acetic acid molecules obtained from proteins and carbohydrates^92^.

## In conclusion

Excessive stressors of all kinds generally generate an oxidative stress, too. In other words, oxidative stress is always an integrated part of other stressors. Low GSH/GSSG ratios indicated that fry tissues were subjected to intensive oxidative stress. Such a ratio could be increased through increasing GSH levels to protect cells against oxidative damage since reduction in GSH levels weaken cell defense mechanisms against reactive oxygen species and ultimately ends up with cell damage an even death. ROS can easily reduce cell GSH levels through lipid peroxidation. Therefore, OH^·^ can generate several lipid hydroperoxide molecules and these molecules in turn greatly impair functions of cell membrane^93^.

The amount of protein during starvation may reflect its abundance in the fry body, their protection by intracellular chaperones or their critical functional roles. Results from the present study showed that starvation stimulates protein synthesis in the *O. mykiss* fry. The results implied a likely correlation between starvation and protein synthesis, which is valuable for investigation. This study have similar result with the several studies reported in fingerlings (*Labeo rohita*)^51^, *Dicentrarchus labrax*^52^ and *Solea senegalensis* during early larval stages^94^.

*Oncorhynchus mykiss* (rainbow trout) is among the most popular sport and market fish. Existing data about *O. mykiss* revealed that dietary lipids and antioxidants with a low molecular weight were quite throughout the initial growth stages, but antioxidant enzymes were dominant in further growth stages^79,95,96^. Present findings revealed that starvation in early stages of development induced oxidative stress in *O. mykiss* fry. Despite the popularity and economic value, the physiology of *O. mykiss* hasn’t been full elucidated, yet. Present findings may have significant contributions to further characterization of oxidative stress in *O. mykiss*. However, further research is still needed about the effects of starvation on physiological mechanisms and oxidative stress response of *O. mykiss* fry.
